# Protocol to generate allorejection-resistant universal CAR-NKT cells and evaluate their efficacy, mechanism of action, safety, and immunogenicity

**DOI:** 10.1016/j.xpro.2025.103810

**Published:** 2025-05-02

**Authors:** Ying Fang, Yuning Chen, Yichen Zhu, Yanxin Tian, Yan-Ruide Li, Lili Yang

**Affiliations:** 1 Department of Microbiology, Immunology & Molecular Genetics, University of California, Los Angeles, Los Angeles, CA 90095, USA; 2 Department of Bioengineering, University of California, Los Angeles, Los Angeles, CA 90095, USA; 3 Molecular Biology Institute, University of California, Los Angeles, Los Angeles, CA 90095, USA; 4 Eli and Edythe Broad Center of Regenerative Medicine and Stem Cell Research, University of California, Los Angeles, Los Angeles, CA 90095, USA; 5 Jonsson Comprehensive Cancer Center, David Geffen School of Medicine, University of California, Los Angeles, Los Angeles, CA 90095, USA; 6 Parker Institute for Cancer Immunotherapy, University of California, Los Angeles, Los Angeles, CA 90095, USA; 7 Goodman-Luskin Microbiome Center, University of California, Los Angeles, Los Angeles, CA 90095, USA

**Keywords:** cancer, immunology, model organisms, biotechnology and bioengineering

## Abstract

Universal chimeric antigen receptor-engineered invariant natural killer T (^U^CAR-NKT) cells overcome the limitations of allogeneic chimeric antigen receptor (CAR)-T cell therapies, such as graft-versus-host disease and allorejection. Here, we present a protocol for generating ^U^CAR-NKT cells from hematopoietic stem and progenitor cells (HSPCs), followed by feeder-free *ex vivo* differentiation. We also outline assays to assess antitumor efficacy, safety, and allorejection resistance.

For complete details on the use and execution of this protocol, please refer to Li et al.^1^

## Highlights

• Generation of ^U^CAR-NKT cells from hematopoietic stem and progenitor cells
• Evaluation of antitumor efficacy, mechanism of action, safety, and immunogenicity
• Steps to conduct assessment in both *in vitro* and *in vivo* models

## Abstract (editor highlights)

Publisher’s note: Undertaking any experimental protocol requires adherence to local institutional guidelines for laboratory safety and ethics.

## Graphical abstract

**Figure:**
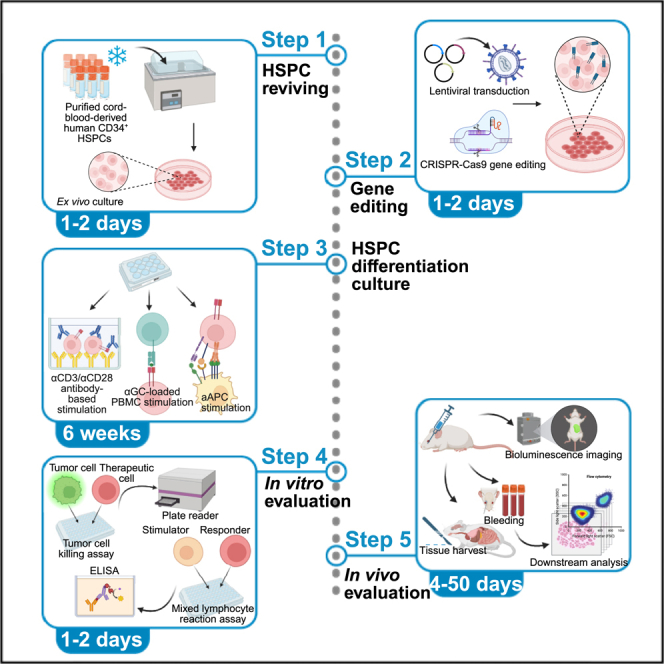

## Before you begin

The clinical-guided method of generating the allogeneic CAR-NKT cells for off-the-shelf cancer immunotherapy with genetically engineered hematopoietic stem and progenitor cells and feeder-free differentiation culture was detailed discussed in the work by Li et al.^2^ In this paper, we shifted the focus on the comprehensive *in vitro* and *in vivo* assays to evaluate the efficacy, mechanism of action, safety, and immunogenicity of the ^U^CAR-NKT (including ^Allo15^BCAR-NKT and ^U15^BCAR-NKT) cells.^1^^,^^3^

For full details on protocol use and execution, please refer to Li et al.^3^

### Institutional permissions

Purified cord-blood-derived human CD34^+^ cells were commercially obtained from the HemaCare. Healthy donor peripheral blood mononuclear cells (PBMCs) were obtained from the UCLA/CFAR Virology Core Laboratory without identification information under federal and state regulations. Primary samples from patients with multiple myeloma (MM) were collected at the Ronald Reagan UCLA Medical Center from consented patients through an IRB-approved protocol (IRB no. 21-001444) and processed. All animal experiments were approved by the Institutional Animal Care and Use Committee of UCLA. All mice were bred and maintained under specific pathogen-free conditions, and all experiments were conducted in accordance with the animal care and use regulations of the Division of Laboratory Animal Medicine at the UCLA.

### Development of humanized mice models

**Timing: 4–20 days**

***Note:*** All mice used in this study are NOD.Cg-Prkdc^scid^ Il2rg^tm1Wjl^/SzJ (NSG) mice, with no prior pre-conditioning. The mice were approximately 6 weeks old at the time of the experiments.

1. Establishing human multiple myeloma-bearing mouse model.
  ***Note:*** This step establishes mouse models harboring human multiple myeloma (MM) cell lines for preclinical testing of ^U^CAR-NKT cells. The following instructions include the intravenous injection of human MM cells.
  a. (Day 0) Inject target MM cells.
    i. Prior to injection, culture the chosen target tumor cell line (e.g., MM.1S-FG, MM.1S-CD1d-FG, ^KO^MM.1S-FG) in R10 medium in a cell culture incubator at 37°C and 5% CO_2_.
      ***Note:*** The optimal confluency of the tumor cell line prior to harvesting is between 60% and 80%. On the day of injection, collect the target tumor cells and resuspend $1\times 10^{6}$ cells in 100 μL of phosphate buffered saline (PBS) for each mouse.
    ii. Use an insulin syringe for tail vein administration.
  b. (Day 0 – Day 4 or Day 20) Monitor mice survival until they receive therapeutic cells or vehicle injection.
    ***Note:*** In low tumor burden model, vehicle and therapeutic cells are injected on Day 4. In high tumor burden model, vehicle and therapeutic cells are injected on Day 20.
    ***Note:*** The vehicle injection consists of a 100 μL dose of PBS.
2. Establishing human ovarian cancer-bearing mouse model.
  ***Note:*** This step generates mouse models bearing human ovarian cancer for preclinical testing of ^U^CAR-NKT cells. The following instructions include the intraperitoneal injection of human ovarian cells.^4^
  a. (Day 0) Inject human ovarian cancer cells.
    i. Prior to injection, culture the chosen target tumor cell line in R10 medium in a cell culture incubator at 37°C and 5% CO_2_.
    ii. On the day of injection, collect the target tumor cells and resuspend $5\times 10^{5}$ cells in 100 μL of PBS for each mouse.
    iii. Use an insulin syringe for intraperitoneal inoculation.
  b. (Day 0 – Day 4) Monitor mice survival until the administration of therapeutic cells or vehicle on Day 4.
3. Establishing donor-mismatched PBMC-engrafted humanized mouse model.
  ***Note:*** This section details the establishment of a model for allorejection studies by injecting NSG mice with donor-mismatched PBMCs. This model facilitates the evaluation of ^U^CAR-NKT cell immunological parameters, including their response to alloreactive immune challenges.
  a. (Day 0) Inject healthy donor-derived, donor-mismatched PBMCs.
    i. Collect and resuspend $1\times 10^{7}$ PBMCs in 100 μL PBS.
      ***Note:*** The injected PBMCs are derived from a single donor distinct from the donors of the therapeutic cells.
    ii. Administer resuspended PBMCs with insulin syringe through tail vein injection.
  b. (Day 0 – Day 4) Monitor mice survival until the administration of therapeutic cells on Day 4.
    ***Note:*** In the donor-mismatched PBMC-engrafted humanized mouse model, therapeutic cells are transduced with Lenti/FG to facilitate BLI analysis. FG refers to a dual-reporter system combining firefly luciferase and enhanced green fluorescent protein, enabling tumor cells engineered with FG to be tracked via luminescence assays and flow cytometry.
    ***Note:*** The protocol of generating lentivirus is described in previous papers.^3^^,^^5^

### Generation of ^U^CAR-NKT cells

**Timing: 6 weeks**

***Note:*** Please refer to Li et al. for details on cell generation and maintenance.^2^ Here, we provide only the key steps for reference to help readers grasp the essential aspects of the procedure.

4. HSPC isolation and preparation.
  a. Obtain human cord blood (CB)-derived CD34^+^ hematopoietic stem and progenitor cells (HSPCs).

**Pause Point:** Cryopreserve and test for purity and viability.

5. Lentivirus production.
  a. Generate lentiviral vectors encoding the chimeric antigen receptor (CAR) of interest and the invariant natural killer T (iNKT) T cell receptor (TCR).
  b. Titrate the lentivirus to determine the optimal multiplicity of infection (MOI).
6. Gene editing for universalization**:** at the HSPC stage, employ CRISPR-Cas9 gene editing to knockout genes responsible for allorejection.

***Note:*** This step is optional for generating allogeneic IL-15-enhanced CAR-engineered NKT (^Allo15^CAR-NKT) cells, but key for ^U15^CAR-NKT cells.

***Note:*** Knock out the *B2M* gene to prevent HLA class I expression, and the *CIITA* gene to prevent HLA class II expression.^1^

7. CAR and iNKT TCR transduction.
  a. Transduce the HSPCs with the lentiviral vectors carrying the CAR and iNKT TCR genes.
    ***Note:*** The HSPCs may or may not have undergone gene editing in step 6.
  b. Incubate for approximately 48 hours at 37°C and 5% CO_2_, and change media.
    ***Note:*** An optional modulation gene (e.g., immune-enhancement or suicide gene) can also be included in the lentiviral vector.
8. *Ex vivo* differentiation culture**.**
  ***Note:*** This step implements a feeder-free, multi-stage culture to differentiate the gene-engineered HSPCs into CAR-NKT cells. This involves five stages over approximately 6 weeks.
  a. HSPC gene engineering (Stage 0): please refer to Steps 6 and 7.
  b. HSPC expansion (Stage 1): Culture in StemSpan SFEM II Medium with StemSpan Lymphoid Progenitor Expansion Supplement.
  c. NKT cell differentiation (Stage 2): Culture in StemSpan SFEM II medium with StemSpan Lymphoid Progenitor Maturation Supplement.
  d. NKT cell deep differentiation (Stage 3): Culture in StemSpan SFEM II medium with StemSpan Lymphoid Progenitor Maturation Supplement and IL-15, with initial activation using ImmunoCult human CD3/CD28/CD2 T Cell Activator.
  e. NKT cell expansion (Stage 4): Employ one of three expansion methods.
    i. αCD3/αCD28 antibody-based.
    ii. α-galactosylceramide (αGC)-loaded healthy donor peripheral blood mononuclear cells (PBMCs).
    iii. Artificial antigen-presenting cells (aAPCs) engineered to express relevant costimulatory molecules and optionally the CAR target antigen. Use NKT cell expansion medium (e.g., CTS OpTmizer or homemade C10 medium) supplemented with IL-7 and IL-15.
9. Cell banking.
  a. After the expansion phase, cryopreserve the resulting ^U^CAR-NKT cells in liquid nitrogen for off-the-shelf use.

## Key resources table

**Table:** 

REAGENT or RESOURCE	SOURCE	IDENTIFIER
**Antibodies**		

Anti-human TCR αβ (clone I26, 1:25 dilution)	BioLegend	CAT#306716, RRID: AB_1953257
Anti-human CD1d (clone 51.1, 1:50 dilution)	BioLegend	CAT#350308, RRID: AB_10642829
Anti-human CD3 (clone HIT3a, 1:500 dilution)	BioLegend	CAT#300308, RRID: AB_314043
Anti-human CD4 (clone OKT4, 1:400 dilution)	BioLegend	CAT#317414, RRID: AB_571959
Anti-human CD8 (clone SK1, 1:300 dilution)	BioLegend	CAT#344714, RRID: AB_2044006
Anti-human CD11b (clone ICRF44, 1:500 dilution)	BioLegend	CAT#301330, RRID: AB_2561703
Anti-human CD14 (clone HCD14, 1:100 dilution)	BioLegend	CAT#325608, RRID: AB_830681
Anti-human CD19 (clone SJ25C1, 1:100 dilution)	BioLegend	CAT#363005, RRID: AB_2564127
Anti-human CD34 (clone 581, 1:500 dilution)	BioLegend	CAT#343505, RRID: AB_1731937
Anti-human CD45 (clone HI30, 1:500 dilution)	BioLegend	CAT#304026, RRID: AB_893337
Anti-human CD45RO (clone UCHL1, 1:100 dilution)	BioLegend	CAT#304206, RRID: AB_2564160
Anti-human CD161 (clone HP-3G10, 1:50 dilution)	BioLegend	CAT#339904, RRID: AB_1501086
Anti-human CD69 (clone FN50, 1:50 dilution)	BioLegend	CAT#310906, RRID: AB_314840
Anti-human BCMA (clone 19F2, 1:50 dilution)	BioLegend	CAT#357506, RRID: AB_2562888
Anti-human CD56 (clone HCD56, 1:10 dilution)	BioLegend	CAT#362545, RRID: AB_2565963
Anti-human CD155 (clone SKII.4, 1:50 dilution)	BioLegend	CAT#337613, RRID: AB_2565746
Anti-human CD163 (clone GHI/61, 1:50 dilution)	BioLegend	CAT#333621, RRID: AB_2563611
Anti-human CD206 (clone 15-2, 1:100 dilution)	BioLegend	CAT#321110, RRID: AB_571885
Anti-human NKp30 (clone P30-15, 1:50 dilution)	BioLegend	CAT#325207, RRID: AB_756111
Anti-human NKG2D (clone 1D11, 1:50 dilution)	BioLegend	CAT#320812, RRID: AB_2234394
Anti-human DNAM-1 (clone 11A8, 1:50 dilution)	BioLegend	CAT#338312, RRID: AB_2561952
Anti-human Granzyme B (clone QA16A02, 1:2,000 or 1:5,000 dilution)	BioLegend	CAT#372208, RRID: AB_2687031
Anti-human Perforin (clone dG9, 1:50 or 1:100 dilution)	BioLegend	CAT#308102, RRID: AB_314700
Anti-human IFN-γ (clone B27, 1:50 dilution)	BioLegend	CAT#506502, RRID: AB_315435
Anti-human NKp46 (clone 9E2, 1:50 dilution)	BioLegend	CAT#331902, RRID: AB_1027637
Anti-human TNF-α (clone MAb11, 1:4,000 dilution)	BioLegend	CAT#502912, RRID: AB_315264
Anti-human HLA-DR (clone L243, 1:250 dilution)	BioLegend	CAT#338312, RRID: AB_2561952
Anti-human IL-2 (clone MQ1-17H12, 1:50 dilution)	BioLegend	CAT#500307, RRID: AB_315093
Human Fc receptor blocking solution (TruStain FcX) (1:100 dilution)	BioLegend	CAT#656112, RRID: AB_2566189
Streptavidin	BioLegend	CAT#405413, RRID: AB_10661733
Anti-human TCR Va24-Jb18 (clone 6B11, 1:20 dilution)	BioLegend	CAT#406421, RRID: AB_2563484
Anti-human mesothelin (MSLN) (clone MN, 1:20 dilution)	BioLegend	CAT#530101, RRID: AB_2571908
Anti-human β2-microglobulin (B2M) (clone 2M2, 1:2,000 or 1:5,000 dilution)	BioLegend	CAT#316312, RRID: AB_10641281
Anti-human MICA/MICB (clone 6D4, 1:100 dilution)	BioLegend	CAT#316312, RRID: AB_10641281
Anti-human NKp44 (clone P44-8, 1:50 dilution)	BioLegend	CAT#325107, RRID: AB_756099
Anti-human ULBP-1 (clone 170818, 1:50 dilution)	R&D Systems	CAT#FAB32652P, RRID: AB_1151946
Goat anti-mouse IgG F(ab’)2 secondary antibody, biotin	R&D Systems	CAT#FAB1380P, RRID: AB_2687471
Anti-human ULBP-2,5,6 (clone 165903, 1:50 dilution)	R&D Systems	CAT#MAB1298
Anti-mouse Fc block (clone 2.4G2, 1:50 dilution)	BD Biosciences	RRID: AB_394656
Anti-human iNKT TCR Vb11 (clone C21, 1:50 dilution)	Beckman Coulter	Product#A66905

**Bacterial and virus strains**		

Lenti/FG	This paper	N/A
Lenti/iNKT-sr39TK	This paper	N/A
Lenti/BCAR	This paper	N/A
Lenti/BCAR-IL15	This paper	N/A
Lenti/iNKT-BCAR-IL-15	This paper	N/A
Lenti/CD1d	This paper	N/A
Lenti/BCMA	This paper	N/A

**Biological samples**		

Human peripheral blood mononuclear cells (PBMCs)	UCLA	N/A
Human cord blood CD34^+^ hematopoietic stem and progenitor cells (HSPCs)	Charles River	N/A

**Chemicals, peptides, and recombinant proteins**		

Recombinant human IL-2	PeproTech	CAT#200–02
Recombinant human IL-3	PeproTech	CAT#200–03
Recombinant human IL-7	PeproTech	CAT#200–07
Recombinant human IL-15	PeproTech	CAT#200–15
Recombinant human Flt3-ligand	PeproTech	CAT#300–19
Recombinant human SCF	PeproTech	CAT#300–07
Recombinant human TPO	PeproTech	CAT#300–18
Recombinant human GM-CSF	PeproTech	CAT#300–03
L-ascorbic acid 2-phosphate	Sigma	CAT#A8960-5G
B-27 supplement (50X), serum-free	Thermo Fisher Scientific	CAT#17504044
α-galactosylceramide (KRN7000)	Avanti Polar Lipids	SKU#867000P-1mg
X-VIVO 15 serum-free hematopoietic cell medium	Lonza	CAT#04–418Q
UltraCULTURE media	Lonza	CAT#BP12725F
RPMI1640 cell culture medium	Corning Cellgro	CAT#10-040-CV
DMEM cell culture medium	Corning Cellgro	CAT#10-013-CV
CTS OpTmizer T cell expansion SFM	Gibco	CAT# A1048501
Fetal bovine serum (FBS)	Sigma	CAT#F2442
MACS BSA stock solution	Miltenyi	CAT#130-091-376
30% BSA	Gemini	CAT#700-110-100
Penicillin-streptomycine-glutamine (P/S/G)	Gibco	CAT#10378016
Penicillin:streptomycin (pen:strep) solution (P/S)	GeminiBio	CAT#400–109
MEM non-essential amino acids (NEAAs)	Thermo Fisher Scientific	CAT#11140050
HEPES buffer solution	Gibco	CAT#15630080
Sodium pyruvate	Gibco	CAT#11360070
Phosphate-buffered saline (PBS) pH 7.4 (1X)	Gibco	CAT#10010–023
β-mercaptoethanol	Sigma	SKU#M6250
Poloxamer Synperonic F108	Sigma	CAT#07579–250G-F
Normocin	InvivoGen	CAT#ant-nr-2
RetroNectin recombination human fibronectin fragment, 2.5 mg	Takara	CAT#T100B
Prostaglandin E2	Cayman Chemical	CAT#14-190-1
Trypan blue solution, 0.4%	Thermo Fisher Scientific	CAT#15250061
Fixable viability dye eFluor 506 Affymetrix	eBioscience	CAT#65-0866-14

**Critical commercial assays**		

Human CD34 MicroBead kit	Miltenyi Biotec	CAT#130-046-703
Fixation/permeabilization solution kit	BD Biosciences	CAT#55474
Foxp3/transcription factor staining buffer set	eBioscience	CAT#00-5523-00
StemSpan lymphoid differentiation coating material (100X)	STEMCELL Technologies	CAT#9925
StemSpan SFEM II	STEMCELL Technologies	CAT#9605
ImmunoCult human CD3/CD28/CD2 T cell activator	STEMCELL Technologies	CAT#10970
TransIT-Lenti transfection reagent	Mirus Bio	CAT#MIR 6600
Amicon Ultra-15 centrifugal filter unit	MilliporeSigma	CAT#UFC910024
CryoStor cell cryopreservation media	Sigma	CAT#C2874-100ML

**Experimental models: Cell lines**		

Human multiple myeloma cell line MM.1S	ATCC	CAT#CRL-2974
Human multiple myeloma cell line MM.1S-FG	This paper	N/A
Human multiple myeloma cell line MM.1S-CD1d-FG	This paper	N/A
Human multiple myeloma cell line ^KO^MM.1S-FG	This paper	N/A
Human ovarian cancer cell line OVCAR8	NIH	N/A
Human ovarian cancer cell line OVCAR8-FG	This paper	N/A

**Experimental models: Organisms/strains**		

NOD.Cg-Prkdc^scid^ Il2rg^tm1Wjl^/SzJ (NSG) mice (6- to 10-week-old female)	UCLA	N/A

**Recombinant DNA**		

Vector: parental lentivector pMNDW	Giannoni et al.^6^; Lan et al.^7^	N/A

**Software and algorithms**		

FlowJo Software 9	FlowJo	https://www.flowjo.com/solutions/flowjo/downloads
Prism 8	GraphPad	https://www.graphpad.com/scientific-software/prism/
Aura imaging software	Spectral Instruments Imaging	https://spectralinvivo.com/software/

**Other**		

MACSQuant analyzer 10 flow cytometer	Miltenyi Biotec	CAT#130-096-343
Infinite M1000 microplate reader	Tecan	CAT#30190085
Drummond Portable Pipet-Aid XP pipet controller	Fisher Scientific	CAT#13-681-06
Corning CoolCell FTS30 freezing container	Corning	CAT#CLS432008
Corning 2 mL internal threaded polypropylene cryogenic vial	Corning	CAT#430488

## Materials and equipment

### C10 medium

This medium is used for the culture of immune cells in this study.

Prepare C10 medium by supplementing RPMI 1640 with 10% fetal bovine serum (FBS), 1% penicillin-streptomycin-glutamine (P/S/G), 1% MEM nonessential amino acids (NEAA), 10 mM HEPES, 1 mM sodium pyruvate, 50 μM β-mercaptoethanol (β-ME), and 100 mg/mL Normocin. All component ratios are volume-to-volume, and all concentrations are final concentrations. To prepare 1000 mL of C10 medium, use an autoclaved 1 L bottle as the sterile container. Attach a 0.22 μm filter top to the bottle and filter the combined components to ensure sterility.

**CRITICAL:** Store the prepared C10 medium at 4 °C in a designated tissue culture refrigerator to maintain stability and prevent contamination.

***Note:*** The medium remains suitable for use for up to one month. Please refer to the table below for precise volumes.

**Table:** 

Reagent	Final concentration	Amount
RPMI	N/A	848 mL
FBS	10%	100 mL
Penicillin-Streptomycin-Glutamine (100X)	1X	10 mL
MEM NEAA (100X)	1X	10 mL
HEPES Buffer Solution (1 M)	0.01 M	10 mL
Sodium Pyruvate (100 mM)	1 mM	10 mL
β-ME (5 mM)	0.05 mM	10 mL
Normocin (500X)	1X	2 mL
**Total**	**N/A**	**1 L**

### R10 medium

This medium is used for the culture of suspension tumor cells in this study.

Prepare R10 medium by supplementing RPMI 1640 with 10% FBS, 1% P/S/G, and 100 mg/mL Normocin. All component ratios are volume-to-volume, and all concentrations are final concentrations. To prepare 1000 mL of R10 medium, use an autoclaved 1 L bottle as the sterile container. Attach a 0.22 μm filter top to the bottle and filter the medium.

**CRITICAL:** Store the prepared R10 medium at 4 °C in a designated tissue culture refrigerator to maintain stability and prevent contamination.

***Note:*** The medium remains suitable for use for up to one month. Please refer to the table below for precise volumes.

**Table:** 

Reagent	Final concentration	Amount
RPMI	N/A	888 mL
FBS	10%	100 mL
Penicillin-Streptomycin-Glutamine (100X)	1X	10 mL
Normocin (500X)	1X	2 mL
**Total**	**N/A**	**1 L**

### D10 medium

This medium is used for the culture of adherent tumor cells in this study.

Prepare D10 medium by supplementing DMEM with 10% FBS, 1% P/S/G, and 100 mg/mL Normocin. All component ratios are volume-to-volume, and all concentrations are final concentrations. To prepare 1000 mL of D10 medium, use an autoclaved 1 L bottle as the sterile container. Attach a 0.22 μm filter top to the bottle and filter the medium.

**CRITICAL:** Store the prepared D10 medium at 4 °C in a designated tissue culture refrigerator to maintain stability and prevent contamination.

***Note:*** The medium remains suitable for use for up to one month. Please refer to the table below for precise volumes.

**Table:** 

Reagent	Final concentration	Amount
DMEM	N/A	888 mL
FBS	10%	100 mL
Penicillin-Streptomycin-Glutamine (100X)	1X	10 mL
Normocin (500X)	1X	2 mL
**Total**	**N/A**	**1 L**

## Step-by-step method details

### *In vitro* assessment of ^U^CAR-NKT cell functionality

**Timing: 1–2 days**

Here, we describe the steps for evaluating the tumor cell-killing efficacy, mechanism of action, safety, and immunogenicity of ^U^CAR-NKT cells *in vitro*.

**Figure 1:**
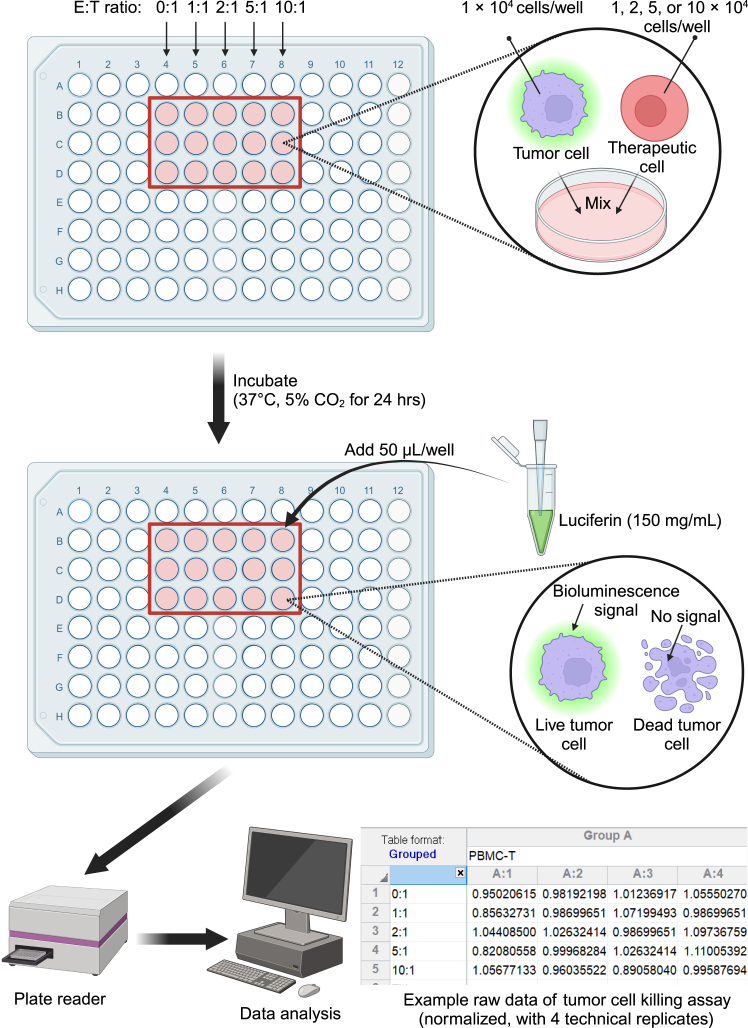
Schematic of tumor cell killing assay setup and data collection Diagram depicting the setup of tumor-killing assays. Firefly luciferase-enhanced GFP-labeled tumor cells (1 x 10^4^) are co-cultured with therapeutic cells at effector-to-target (E:T) ratios of 1:1, 2:1, 5:1, or 10:1. After 24 hours, luciferin is added, and bioluminescence is quantified using a plate reader to assess tumor cell viability. A representative tumor-killing assay raw dataset is included in the figure for reference. This example features five E:T ratios as described, along with the PBMC-T group, each performed in four technical replicates.

**Figure 2:**
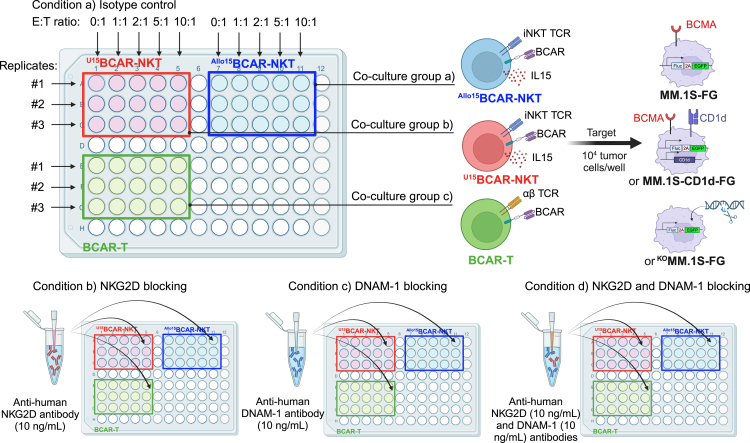
Plate layout for *in vitro* mechanism of action studies Experimental plate setup for investigating the *in vitro* mechanism of action of ^U^CAR-NKT cells. Three human multiple myeloma (MM) cell lines (MM.1S-FG, MM.1S-CD1d-FG, KOMM.1S-FG) are co-cultured with therapeutic cells under four conditions: isotype control, NKG2D blocking, DNAM-1 blocking, and NKG2D/DNAM-1 double blocking.

**Figure 3:**
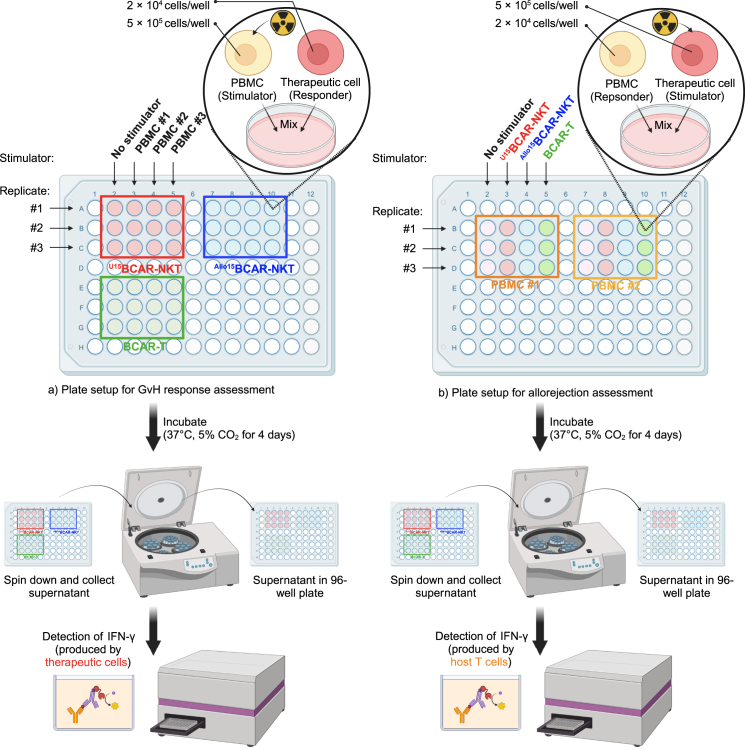
Schematics and plate layout for *in vitro* mixed lymphocyte reaction assay Schematic of the mixed lymphocyte reaction (MLR) assay used to assess graft-versus-host effects and T cell-mediated allorejection *in vitro*. Co-cultured cells are incubated for four days before ELISA-based quantification of IFN-γ production as a measure of immune activation. GvH, graft-versus-host.

1. Assessing of tumor-killing efficacy.
  ***Note:*** This step evaluates the cytotoxic potential of ^U^CAR-NKT cells using co-culture assays with target tumor cell lines.
  a. (Day 0) Prepare target tumor cells.
    i. Culture the chosen target tumor cell line (e.g., MM.1S-FG, MM.1S-CD1d-FG, ^KO^MM.1S-FG) in R10 medium in a cell culture incubator at 37°C and 5% CO_2_.
      ***Note:*** These cell lines are genetically engineered to express fluorescent or luminescent reporters for easy quantification. MM.1S-CD1d-FG is an MM.1S-FG cell line further engineered to overexpress human CD1d. ^KO^MM.1S-FG is an MM.1S-FG cell line further engineered to knock out BCMA and CD1d using CRISPR-Cas9.
      ***Note:*** It is ideal to set up the killing assay when the tumor cells reach ∼80–90% confluency.
  b. (Day 0) Prepare therapeutic cells.
    i. Culture the ^U15^BCAR-NKT cells, ^Allo15^BCAR-NKT cells, BCAR-T cells and PBMC-T cells separately in C10 medium.
      ***Note:*** Please refer to Li et al. for details on cell generation and maintenance.^2^
  c. (Day 1) Set up co-culture assay.
    i. Harvest the target tumor cells and therapeutic cells and resuspend them in C10 medium.
      **CRITICAL:** Keep the cells on ice or at 4°C after thawing and resuspension to preserve viability. Rapid temperature fluctuations or prolonged exposure to higher temperatures can compromise cell integrity and function. Minimize handling time at room temperature and proceed immediately to downstream processing to maintain optimal viability.
    ii. Prepare a 96-well clear flat-bottom black plate.
    iii. Seed 1 × 10^4^ tumor cells per well in 100 μL of C10 medium.
    iv. Add therapeutic cells to the designated wells at varying effector cell-to-target cell (E:T) ratios.
      ***Note:*** The therapeutic cells are ^U15^BCAR-NKT cells, ^Allo15^BCAR-NKT cells, BCAR-T cells, or PBMC-T cells.
      ***Note:*** The E:T ratios could be 0:1, 1:1, 2:1, 5:1, 10:1.
    v. Adjust the volume of the therapeutic cell suspension to maintain a consistent final volume of 200μL in each well.
    vi. Add αGC (at a final concentration of 100 ng/mL) to wells containing MM.1S-CD1d-FG to stimulate iNKT TCR activation.
    vii. Incubate the plate in a cell culture incubator at 37°C and 5% CO_2_ for 24 hours. Troubleshooting 1.
      **CRITICAL:** Include ∼3–5 technical replicates per condition. Due to edge effects, media in the outermost co-culture wells may evaporate more quickly, leading to variability and potential inaccuracies. To minimize this, avoid using edge wells or implement a consistent plate setup. Please refer to Figure 1 for a detailed plate setup.
  d. (Day 2) Quantification of tumor cell killing.
    i. After 24 hours of co-culture, add 100 μL of 150 mg/mL D-luciferin to each well, and incubate for 5 minutes.
    ii. Measure the luciferase activity using an Infinite M1000 microplate reader (Tecan).
      ***Note:*** For luciferase quantification, the default LUM setting on the Tecan reader is recommended, though customized settings can be used as needed.
    iii. Analyze the data to calculate the percentage of tumor cell killing for each E:T ratio and compare the killing efficacy of different therapeutic cells.
      ***Note:*** The luminescence signal is proportional to the number of live tumor cells. Reduced luminescence indicates tumor cell killing.
2. Assessment of mechanism of action.
  ***Note:*** This step analyzes tumor-targeting mechanisms using iNKT TCR-, CAR-, and NKR-mediated pathways.
  a. (Day 0) Prepare target tumor cells and therapeutic cells.
    i. Follow Section 1. a. (Assessing of tumor-killing efficacy: Prepare target tumor cells) and 1. b. (Assessing of tumor-killing efficacy: Prepare therapeutic cells).
  b. (Day 1) Set up co-culture assay.
    i. Follow Section 1. c. (Assessing of tumor-killing efficacy: Set up co-culture assay) Step i.-iv. for initial set up.
    ii. Set up three co-culture groups: ^KO^MM.1S-FG tumor only, ^KO^MM.1S-FG + ^U^15BCAR-NKT, and ^KO^MM.1S-FG + ^Allo15^BCAR-NKT cells.
  c. (Day 1) Study iNKT TCR engagement.
    i. Compare the killing efficacy with groups in Section 1. c.
      ***Note:*** iNKT TCR engagement is assessed by comparing ^U^CAR-NKT cells versus BCAR-T cells tumor cell killing efficacy.
      ***Note:*** Please refer to Figure 2 for detailed instructions on comparison analysis.
  d. (Day 1) Study CAR engagement.
    i. Compare the killing efficacy with groups in Section 1. c.
      ***Note:*** CAR engagement is assessed by comparing ^U^CAR-NKT cells and BCAR-T cells versus PBMC-T tumor cell killing efficacy.
      ***Note:*** Please refer to Figure 2 for detailed instructions on comparison analysis.
  e. (Day 1) Study NKR engagement.
    i. Include the following conditions for each experimental group from Section 2: isotype control (at a final concentration of 10 μg/mL), anti-human NKG2D antibody (at a final concentration of 10 μg/mL), anti-human DNAM-1 antibody (at a final concentration of 10 μg/mL), and anti-human NKG2D and anti-human DNAM-1 antibody (both at a final concentration of 10 μg/mL)
      ***Note:*** Please refer to Figure 2 for a detailed plate setup.
  f. (Day 2) Quantification of tumor cell killing.
    i. Follow Section 1. d. (Assessing of tumor-killing efficacy: Quantification of tumor cell killing).
3. Assessment of safety.
  ***Note:*** This step studies the graft-versus-host (GvH) response of ^Allo/U15^BCAR-NKT cells. An *in vitro* mixed lymphocyte reaction (MLR) assay is used.
  a. (Day 1) Prepare stimulator cells.
    i. Isolate peripheral blood mononuclear cells (PBMCs) from healthy donors and resuspend in C10 medium.
    ii. Load αGC (at a final concentration of 100 ng/mL) to the PBMCs.
      ***Note:*** This step is optimal. αGC-loaded PBMCs can be used to study the GvH response in the presence of an NKT agonist, whereas PBMCs without αGC can be used to study the GvH response under baseline conditions. Please refer to Li et al. for detailed instruction on loading αGC to PBMCs.^3^
      **CRITICAL:** Keep the cells on ice or at 4°C after thawing and resuspension to preserve viability. Ensure the cells remain on ice during irradiation. Rapid temperature fluctuations or prolonged exposure to higher temperatures can compromise cell integrity and function. Minimize handling time at room temperature and proceed immediately to downstream processing.
    iii. Irradiate the PBMCs at 2,500 rads for 30 minutes.
      **CRITICAL:** Various irradiation systems, such as X-ray devices, gamma sources, or linear accelerators, can be utilized for this process. Adhering to strict safety guidelines is essential to protect researchers and preserve experimental reliability when handling irradiation equipment.
  b. (Day 1) Prepare responder cells.
    i. Resuspend the responder cells in C10 medium.
      ***Note:***^Allo15^BCAR-NKT or ^U15^BCAR-NKT cells will serve as responder cells. PBMC-derived BCAR-T cells can be included as a responder control.
  c. (Day 1) Set up MLR assay.
    i. Prepare a 96-well round-bottom plate.
    ii. Seed 5 × 10^5^ stimulator PBMCs per well in 100 μL of C10 medium.
    iii. Seed 2 × 10^4^ responder cells per well in 100 μL of C10 medium. Adjust the volume of the therapeutic cell suspension to maintain a consistent final volume of 200μL in each well.
    iv. Incubate the plate in a cell culture incubator at 37°C and 5% CO_2_ for 4 days. Troubleshooting 1.
      **CRITICAL:** Include ∼3–5 technical replicates per condition. Due to edge effects, media in the outermost co-culture wells may evaporate more quickly, leading to variability and potential inaccuracies. To minimize this, avoid using edge wells or implement a consistent plate setup. Please refer to Figure 3 for a detailed plate setup.
  d. (Day 4) Measure IFN-γ production.
    i. Collect the cell culture supernatants from the 96-well plate.
    ii. Perform an enzyme-linked immunosorbent cytokine assay (ELISA) to measure IFN-γ production.
      ***Note:*** The production of IFN-γ will be solely attributed to the ^Allo15^BCAR-NKT or ^U15^BCAR-NKT or PBMC-derived BCAR-T responder cells.
4. Assessment of Immunogenicity.
  ***Note:*** This step studies the potential for ^Allo/U15^BCAR-NKT cells to be recognized and targeted by host T cells, and an *in vitro* MLR assay is used.
  a. (Day 1) Prepare stimulator cells.
    i. Harvest stimulator cells and resuspend in C10 medium.
    ii. Irradiate the stimulator cells at 2,500 rads.
      ***Note:***^Allo15^BCAR-NKT or ^U15^BCAR-NKT cells will serve as stimulator cells.
      **CRITICAL:** Keep the cells on ice or at 4°C after thawing and resuspension to preserve viability. Ensure the cells remain on ice during irradiation. Rapid temperature fluctuations or prolonged exposure to higher temperatures can compromise cell integrity and function. Minimize handling time at room temperature and proceed immediately to downstream processing.
  b. (Day 1) Prepare responder cells.
    i. Isolate PBMCs from healthy donors and resuspend in C10 medium.
  c. (Day 1) Set up MLR assay.
    i. Prepare a 96-well round-bottom plate.
    ii. Seed 5 × 10^5^ irradiated stimulator cells (either ^Allo15^BCAR-NKT or ^U15^BCAR-NKT cells) per well in 100 μL of C10 medium.
    iii. Seed 2 × 10^4^ responder cells (PBMCs) per well in 100 μL of C10 medium. Adjust the volume of the therapeutic cell suspension to maintain a consistent final volume of 200 μL in each well.
    iv. Incubate the plate in a cell culture incubator at 37°C and 5% CO_2_ for 4 days. *Troubleshooting 1.*
      ***Note:*** This setup is used to study T-cell-mediated allorejection. To study NK cell–mediated allorejection, co-culture PBMC-derived NK cells with unirradiated therapeutic cells under the same conditions and using the same cell numbers as in the T cell–mediated allorejection assay.^1^
      ***Note:*** For NK cell-mediated allorejection, direct cytotoxicity against therapeutic cells should be assessed using flow cytometry at the end of the co-culture. *Troubleshooting 2*.
      **CRITICAL:** Include ∼3–5 technical replicates per condition. Due to edge effects, media in the outermost co-culture wells may evaporate more quickly, leading to variability and potential inaccuracies. To minimize this, avoid using edge wells or implement a consistent plate setup. Please refer to Figure 3 for a detailed plate setup.
  d. (Day 4) Measure IFN-γ production.
    i. Collect the cell culture supernatants from the 96-well plate.
    ii. Perform an ELISA to measure IFN-γ production.
      ***Note:*** In T cell-mediated allorejection studies, IFN-γ production is attributed to PBMC responder cells.
      ***Note:*** In NK cell-mediated allorejection studies, killing of therapeutic cells is attributed to PBMC-NK cells.

### *In vivo* functional and safety studies

**Timing: 4–50 days**

Here, we describe the steps for evaluating the tumor cell-killing efficacy, mechanism of action, safety, and immunogenicity of ^U^CAR-NKT cells *in vivo*.

**Figure 4:**
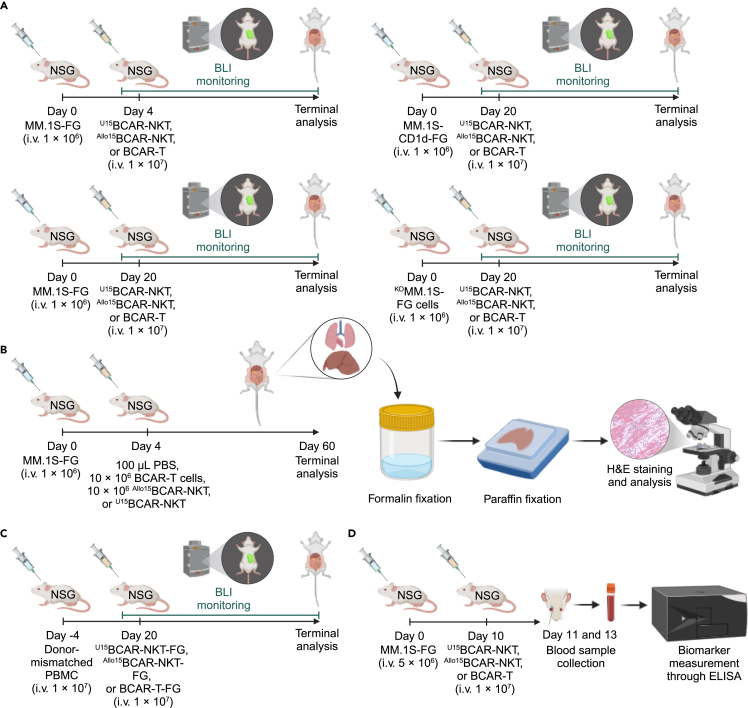
Experimental design for *in vivo* GvH risk and allorejection assessment (A) Schematic of tumor cell killing efficacy and mechanism of action evaluation. (B) Experimental setup for assessing the GvHD risk associated with ^U^CAR-NKT cells. (C) Schematic of T cell-mediated allorejection assessment of ^U^CAR-NKT cells. (D) Experimental design for evaluating CRS-like responses induced by ^U^CAR-NKT cells.

5. Assessment of Tumor Killing Efficacy.
  ***Note:*** This step evaluates the *in vivo* antitumor efficacy and pharmacokinetics of ^U^CAR-NKT using NOD.Cg-Prkdc^SCID^Il2rg^tm1Wjl^/SzJ (NOD/SCID/IL-2Rγ^−/−^, NSG) xenograft mice injected with human MM cell lines, including MM.1S-FG, MM.1S-CD1d-FG, and ^KO^MM.1S-FG cells.
  a. (Day 0) Tumor cell inoculation.
    i. Intravenously inject NSG mice with one of the human MM cell lines from tail vein, including MM.1S-FG, MM.1S-CD1d-FG, and ^KO^MM.1S-FG cells (1 × 10^6^ cells per mouse). Troubleshooting 4.
      ***Note:*** These three cell lines are used for the following conditions: MM.1S-FG for both low and high tumor burdens and ^KO^MM.1S-FG for low tumor burden only.
      ***Note:*** For detailed model development, please refer to Section development of humanized mice models.
  b. (Day 4 or Day 20) Therapeutic cell administration.
    ***Note:*** Administer treatments retro-orbitally on Day 4 or Day 20.
    ***Note:*** Injection on Day 4 is for the low tumor burden models, and injection on Day 20 is for the high tumor burden models.
    ***Note:*** These time points were selected to mimic different stages of tumor progression.
    ***Note:*** Administer treatments via retro-orbital intravenous injection to the mice within each group injected with a human MM cell line.
    i. Divide the mice are divided into the following groups: vehicle group (100 μL PBS per mouse), control group (10 × 10^6^ BCAR-T cells in 100 μL PBS per mouse), and experimental group (10 × 10^6 Allo15^BCAR-NKT or ^U15^BCAR-NKT cells 100 μL PBS per mouse). Troubleshooting 5.
  c. (Day 4 or Day 20) Monitor mice and data collection. Monitor survival and measure tumor burden twice per week using bioluminescence imaging until euthanasia criteria are met for each mouse. Troubleshooting 3.
    i. For each mouse, intraperitoneally inject 1 mg D-Luciferin (dissolved in 100 μL PBS) to visualize tumor cells.
    ii. Five minutes later, measure total body bioluminescence using a Spectral Advanced Molecular Imaging HTX imaging system (Spectral Instrument Imaging).
  d. (Day 50) Data analysis.
    i. Export the imaging data using the AURA imaging software (Spectral Instrument Imaging, version 3.2.0).
    ii. There are multiple ways to analyze the data, including: quantify the total bioluminescence level (p/s) of your region of interest, look at the distribution of the luminescence in the mouse organs, and record the Kaplan Meier survival curve.
    iii. Compare the effect of each treatment on tumor load and survival rate within each tumor cell line group to assess treatment-specific antitumor efficacy.
6. Assessment of mechanism of action.
  ***Note:*** This step evaluates the tumor-targeting mechanisms of ^U^CAR-NKT cells *in vivo*, including CAR-, iNKT TCR-, and NKR-mediated pathways, using NSG xenograft mice engrafted with human MM.1S-FG, MM.1S-CD1d-FG, and ^KO^MM.1S-FG cell lines.
  a. (Day 0) Tumor cell inoculation.
    i. Follow Section 1. a. of development of humanized mice models.
  b. (Day 4 or Day 20) Therapeutic cell administration.
    ***Note:*** Administer treatments via retro-orbital intravenous injection to the mice within each group injected with a human MM cell line.
    i. Divide the mice are divided into four groups.
      ***Note:*** Tumor-free group (100 μL PBS per mouse without prior tumor inoculation from step a), vehicle group (100 μL PBS per mouse), control group (10 × 10^6^ BCAR-T cells in 100 μL PBS per mouse), and experimental group (10 × 10^6 Allo15^BCAR-NKT or ^U15^BCAR-NKT cells 100 μL PBS per mouse).
  c. (Day 4 or Day 20) Monitor mice and data collection.
    i. Monitor survival and measure tumor burden twice per week using bioluminescence imaging until euthanasia criteria are met for each mouse. Troubleshooting 3.
      ***Note:*** Follow Section 1. c. of development of humanized mice models.
  d. (Day 50) Compare the effect of the same treatment on tumor load and survival rate across all tumor cell line groups to assess pathway-specific tumor-targeting mechanisms.
    i. CAR-mediated pathway: MM.1S-FG cells.
    ii. iNKT TCR-mediated pathway: MM.1S-CD1d-FG cells.
    iii. NKR-mediated pathway: ^KO^MM.1S-FG cells.
      ***Note:*** Please refer to Figure 4 for detailed instructions on comparison analysis.
7. Assessment of graft-versus-host disease (GvHD).
  ***Note:*** This step evaluates the *in vivo* safety profile/GvHD of ^U^CAR-NKT cells using NSG xenograft mice inoculated with the MM.1S-FG cell line.
  a. (Day 0) Tumor cell inoculation.
    i. Intravenously inject NSG mice with human MM.1S-FG cells (1 × 10^6^ cells per mouse in 100 μL PBS).
  b. (Day 4) Therapeutic cell administration.
    i. Administer treatments via retro-orbital intravenous injection to the mice injected with a human MM cell line. And the mice are divided into the following groups: tumor-free group (100 μL PBS per mouse without prior tumor inoculation from step a), vehicle group (100 μL PBS per mouse), control group (10 × 10^6^ BCAR-T cells in 100 μL PBS per mouse), and experimental group (10 × 10^6 Allo15^BCAR-NKT or ^U15^BCAR-NKT cells 100 μL PBS per mouse).
  c. (Day 50) On the day of euthanasia, perform terminal tissue harvest based on the selected model.
    i. After isolating organs, fix tissues in 10% neutral buffered formalin for up to 36 hours.
    ii. Embed fixed tissues in paraffin and section at 5 μm thickness.
  d. (Day 54) After fixation, perform H&E staining following a standard protocol or outsource to an external agency.
  e. (Day 54) After H&E staining, image the stained tissue sections for GvHD assessment.
    i. Use an Olympus BX51 upright microscope equipped with an Optronics Macrofire CCD camera (AU Optronics) to image the stained section.
    ii. Analyze the images with the Optronics PictureFrame software (AU Optronics).
    iii. Compare the immune cell infiltration area between different groups to assess GvHD.
8. Assessment of immunogenicity.
  ***Note:*** This step evaluates the immunogenicity of ^U^CAR-NKT cells, including host T cell-mediated allorejection.
  a. Evaluation of T cell-mediated allorejection.
    i. (Day −4) Intravenously inject NSG xenograft mice with 1 × 10^7^ donor-mismatched PBMCs.
    ii. (Day 0) Administer treatments via retro-orbital intravenous injection to the mice within each group injected with donor-mismatched PBMCs. And the mice are divided into the following groups: control group (10 × 10^6^ BCAR-T cells in 100 μL PBS per mouse) and experimental group (10 × 10^6 Allo15^BCAR-NKT or ^U15^BCAR-NKT cells 100 μL PBS per mouse).
    iii. Follow Section 5. c. to monitor therapeutic cell progression and survival rates. Change the amount of D-Luciferin to 3 mg/mouse therapeutic cell visualization.
    iv. Follow Section 5. d. for data analysis.
9. Assessment of CRS toxicity.
  a. Evaluate the CRS-like response associated with ^U^CAR-NKT cells.
    i. (Day 0) Intravenously inject NSG xenograft mice with 5 × 10^6^ MM.1S-FG cells per mouse in 100 μL PBS.
    ii. (Day 10) Administer treatments to mice via intravenous injection.
      ***Note:*** Follow Section 5. b. for details on the therapeutic cell peparation and the treatment dosages assigned to each group.
    iii. (Day 11 and 13) collect blood samples from the mice. Measure CRS-related biomarkers such as mouse IL-6 and SAA-3 in serum through ELISA.
      ***Note:*** Measure mouse IL-6 using paired purified anti-mouse IL-6 antibody and biotin anti-mouse IL-6 antibody (BioLegend), and measure mouse SAA-3 using the Mouse SAA-3 ELISA kit (MilliporeSigma), following the manufacturer’s instructions.
    iv. Data analysis: compare IL-6 and SAA-3 secretion levels to evaluate the CRS-like response.

## Expected outcomes

This protocol enables the generation and functional evaluation of HSPC-derived ^U15^BCAR-NKT and ^Allo15^BCAR-NKT cells, demonstrating their potential as an effective allogeneic cell therapy platform. The antibodies listed in the key resources table are utilized for phenotypic characterization and functional evaluation of the therapeutic cells, as described in the published work by Li et al.^1^ These antibodies are also employed in assessing the expected outcomes outlined in this protocol.

**Figure 5:**
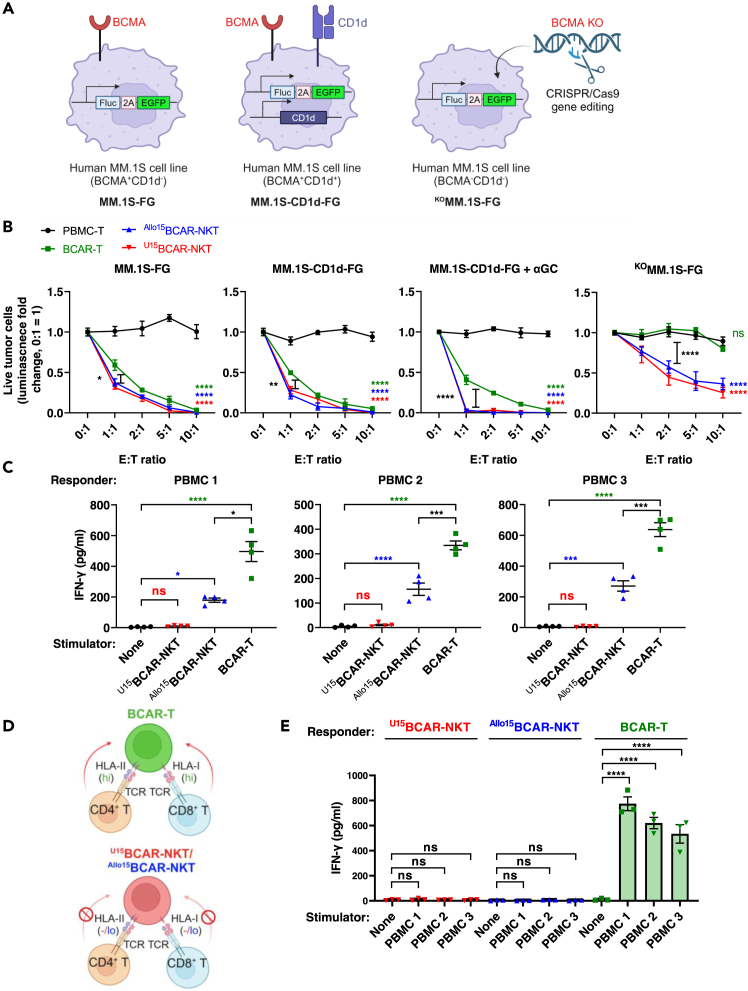
Expected outcomes of *in vitro* tumor cell killing and mixed lymphocyte reaction assays (A) Schematic representation of the generation of three human MM cell lines. (B) Tumor cell killing efficacy of ^U^CAR-NKT cells at 24 hours (n = 4), with conventional BCAR-T cells included as a benchmark control. (C) Assessment of T cell-mediated allorejection against ^U^CAR-NKT cells using an *in vitro* MLR assay. PBMCs from ≥ 3 randomly mismatched healthy donors serve as responder cells, while irradiated ^U^CAR-NKT cells function as stimulators. BCAR-T cells are included as an allorejection control. IFN-γ production is quantified by ELISA on day 4 (n = 4). (D) Schematic depicting the HLA-low/negative phenotype of ^U^CAR-NKT cells and their resistance to T cell-mediated allorejection. (E) ELISA quantification of IFN-γ production on day 4. N, no addition of stimulator PBMCs (n = 3). Data are representative of >5 independent experiments and presented as mean ± SEM. Statistical significance was determined by one-way ANOVA (C and E) or two-way ANOVA (B); ns, not significant; ∗p < 0.05; ∗∗p < 0.01; ∗∗∗p < 0.001; ∗∗∗∗p < 0.0001.

In *in vitro* functional assays, ^U15^BCAR-NKT and ^Allo15^BCAR-NKT cells exhibited comparable tumor-killing capacity, displaying enhanced cytotoxicity against BCMA-expressing tumor cells relative to BCAR-T cells (Figures 5A and 5B). Notably, even in the absence of BCMA expression, ^U15^BCAR-NKT and ^Allo15^BCAR-NKT cells maintained potent, dose-dependent tumor clearance, underscoring their superior adaptability in targeting malignant cells (Figures 5A and 5B).

To assess their susceptibility to host immune rejection, a MLR assay was conducted to evaluate T cell-mediated alloreactivity. Unlike BCAR-T cells, ^U15^BCAR-NKT and ^Allo15^BCAR-NKT cells exhibited significantly lower IFN-γ production, indicating reduced recognition and rejection by host T cells due to their low expression of HLA class I and II molecules (Figures 5C and 5D). Furthermore, CRISPR-Cas9-mediated knockout of HLA class I and II molecules in ^U15^BCAR-NKT cells at the HSPC stage further reduced alloreactivity compared to ^Allo15^BCAR-NKT cells, highlighting the potential of genetic modifications to enhance immune compatibility (Figure 5C).

To assess their risk of GvHD, a second MLR assay was performed to evaluate their response to host cells. ^U15^BCAR-NKT and ^Allo15^BCAR-NKT cells exhibited minimal IFN-γ secretion following PBMC stimulation, in stark contrast to BCAR-T cells, which elicited a strong IFN-γ response (Figure 5E). These findings indicate that ^U15^BCAR-NKT and ^Allo15^BCAR-NKT cells pose a significantly lower risk of inducing GvH reactions, reinforcing their feasibility for allogeneic applications.

Collectively, these results establish ^U15^BCAR-NKT and ^Allo15^BCAR-NKT cells as promising off-the-shelf immunotherapies with effective tumor targeting, minimized immune rejection, and broad applicability in adoptive cell therapy. This protocol provides a scalable approach for generating HSPC-derived ^U15^BCAR-NKT cells and facilitates the evaluation of their efficacy, mechanism of action, safety, and immunogenicity.

## Quantification and statistical analysis

A Prism 8 software (GraphPad) was utilized for all statistical analysis. Pairwise comparisons were performed with a 2-tailed Student’s t test. Multiple comparisons were performed with an ordinary 1-way ANOVA, followed by Tukey’s multiple comparisons test. Unless otherwise indicated, data are presented as the mean GSEM. In all figures and figure legends, ‘‘n’’ represents the number of biological replicates in which the experiment was performed. A P value of less than 0.05 was considered significant. ns, not significant; ∗p < 0.05; ∗∗p < 0.01; ∗∗∗p < 0.001; ∗∗∗∗p < 0.0001.

## Limitations

*In vitro* studies provide insights into the mechanism of action but are inherently artificial and lack the complexity of physiological systems. *In vivo* humanized mouse models offer a more comprehensive and physiologically relevant platform to evaluate therapeutic efficacy and safety. However, these models still fail to fully recapitulate the intricacies of the tumor microenvironment present in human patients.

## Troubleshooting

### Problem 1

When performing the killing or MLR assay, cells in the outermost wells often exhibit reduced viability.

### Potential solution

This is attributed to the “edge effect,” typically caused by increased media evaporation in these wells. To mitigate this issue, fill the outermost wells with C10 medium only, leaving inner wells for experimental conditions. This approach minimizes media evaporation and protects the viability of cells in the experimental wells.

### Problem 2

Flow cytometry data may exhibit out-of-gate or inaccurate population distributions, compromising the reliability of results.

### Potential solution

This issue often arises from suboptimal compensation settings, where fluorescence spillover between channels is not adequately corrected. To address this, optimize compensation settings using appropriate single-stain controls that match the experimental fluorophores and antibody panel. Perform compensation setup with freshly prepared samples under identical staining and acquisition conditions.

### Problem 3

NSG mice frequently exhibit CAR-T cell-related CRS-like symptoms, including severe weight loss, which can result in premature death, compromise experimental outcomes, and raise ethical concerns.

### Potential solution

Mice should be monitored daily for health and clinical symptoms.^8^ Prompt intervention is necessary upon observing CRS-like signs. If weight loss exceeds 20% of the baseline, humane euthanasia is recommended to prevent unnecessary suffering while maintaining ethical standards.

### Problem 4

Improper tail vein injections during tumor inoculation can result in failed delivery of tumor cells to the bloodstream, compromising model development.

### Potential solution

Accurate injection technique is crucial. Use an insulin syringe for tumor cell injection, ensuring the mouse’s tail is fully extended and aligned horizontally with the ground. The injection angle should approach 180 degrees to optimize vein access.

### Problem 5

Inadequate retro-orbital injections can occur when therapeutic cells are not properly delivered into the vein, reducing model success.

### Potential solution

To ensure successful retro-orbital injections, avoid visible bleeding or cell leakage from the mouse eye. The insulin syringe needle should penetrate into the retro-orbital sinus, ensuring accurate cell delivery without over-insertion.

## Resource availability

### Lead contact

Further information and requests for resources and reagents should be directed to and will be fulfilled by the lead contact, Lili Yang (liliyang@ucla.edu).

### Technical contact

Technical questions on executing this protocol should be directed to and will be answered by the technical contact, Yan-Ruide Li (charlie.li@ucla.edu).

### Materials availability

This study did not generate new unique reagents.

### Data and code availability

This paper does not report original code.

Any additional information required to reanalyze the data reported in this working paper is available from the lead contact upon request.

## Acknowledgments

We thank the University of California, Los Angeles (UCLA) CFAR Virology Core for providing human cells and the UCLA BSCRC Flow Cytometry Core Facility for cell sorting support. This work was supported by a Partnering Opportunity for Discovery Stage Award from the California Institute for Regenerative Medicine (DISC2-13505 to L.Y.), a UCLA BSCRC Stem Cell Research Innovation Award (to L.Y.), and an Ablon Scholars Award (to L.Y.). L.Y. is an investigator of the Parker Institute for Cancer Immunotherapy (PICI) at UCLA. Y.-R.L. is a postdoctoral fellow supported by a UCLA MIMG M. John Pickett Post-Doctoral Fellow Award, a CIRM-BSCRC Postdoctoral Fellowship, a UCLA Sydney Finegold Postdoctoral Award, and a UCLA Chancellor’s Award for Postdoctoral Research.

Graphical abstract/figures were created using Biorender.com.

## Author contributions

Y.F., Y.C., Y.-R.L., and L.Y. designed the experiments, analyzed the data, and wrote the manuscript. Y.-R.L. and L.Y. conceived and oversaw the study, with assistance from Y.F. and Y.C. Y.F. and Y.C. performed experiments and wrote the manuscript, with assistance from Y.Z. and Y.T.

## Declaration of interests

Y.-R.L. and L.Y. are inventors on patents relating to this manuscript. L.Y. is a scientific advisor to AlzChem and Amberstone Biosciences and a co-founder, stockholder, and advisory board member of Appia Bio. None of the declared companies contributed to or directed any of the writing of this manuscript.
